# Thermoresponsive and Conductive Chitosan-Polyurethane Biocompatible Thin Films with Potential Coating Application

**DOI:** 10.3390/polym13030326

**Published:** 2021-01-20

**Authors:** Junpeng Xu, Chih-Yu Fu, Yu-Liang Tsai, Chui-Wei Wong, Shan-hui Hsu

**Affiliations:** 1 Institute of Polymer Science and Engineering, National Taiwan University, No. 1, Sec. 4 Roosevelt Road, Taipei 10617, Taiwan; f07549033@ntu.edu.tw (J.X.); r07549005@ntu.edu.tw (C.-Y.F.); jimmy821212@gmail.com (Y.-L.T.); caryn1111@gmail.com (C.-W.W.); 2 Institute of Cellular and System Medicine, National Health Research Institutes, No. 35 Keyan Road, Miaoli 35053, Taiwan

**Keywords:** conductive thin film, thermoresponsive, polyurethane, chitosan, biocompatibility, polymer coating

## Abstract

Conductive thin films have great potential for application in the biomedical field. Herein, we designed thermoresponsive and conductive thin films with hydrophilicity, strain sensing, and biocompatibility. The crosslinked dense thin films were synthesized and prepared through a Schiff base reaction and ionic interaction from dialdehyde polyurethane, N-carboxyethyl chitosan, and double-bonded chitosan grafted polypyrrole. The thin films were air-dried under room temperature. These thin films showed hydrophilicity and conductivity (above 2.50 mS/cm) as well as responsiveness to the deformation. The tensile break strength (9.72 MPa to 15.07 MPa) and tensile elongation (5.76% to 12.77%) of conductive thin films were enhanced by heating them from 25 °C to 50 °C. In addition, neural stem cells cultured on the conductive thin films showed cell clustering, proliferation, and differentiation. The application of the materials as a conductive surface coating was verified by different coating strategies. The conductive thin films are potential candidates for surface modification and biocompatible polymer coating.

## 1. Introduction

Conductive polymers are considered to be a class of organic materials having electric and optical properties. The conductivity of such materials comes from different types of electric charges, which are the conjugated π system, conductive nanomaterials, conductive polymers, and ions [1,2]. Among different forms of conductive polymers, conductive films hold the advantages of having short processing time, low manufacturing price, and industrially scalable potential [3,4,5]. Moreover, conductive films are versatile so they can be designed and applied in biosensors, flexible electronics, neural regeneration, antifouling coating, wearable electronics, and optoelectronic devices [4,6,7,8,9,10], depending on various selections of materials. Meanwhile, the functionalization of a few common film-forming materials (e.g., polyurethane [11] and chitosan [12]) may be the key to a breakthrough of the preparation for multifunctional films.

Polyurethanes, consisting of hard and soft segments, are a class of polymeric materials with tunable mechanical properties, firm chemical resistance, and stable heat insulation [3,13,14]. Biodegradable waterborne polyurethanes, also possessing biocompatibility, are of particular interest for their environmentally friendly process and impact [3,15]. Polyurethane films, as a kind of classic products, have a wide range of applications, including for gas barriers, flame retardants, electromagnetic interference shielding, and nerve conduits [13,14,16,17]. Additionally, polyurethanes can be modified to have different functionalities by changing the soft segments or end groups, making polyurethanes thermoresponsive, photosensitive, or crosslinkable [18,19,20]. A crosslinkable waterborne polyurethane, dialdehyde polyurethane (DAPU), has been developed recently to broaden the horizon for combining synthetic polymers with natural polymers [20]. With the above-mentioned development, the applications of polyurethane-based materials are expected to integrate more functions together and be further expanded from classic synthetic polymers to multifunctional biomedical applications.

Chitosan is a linear and cationic polysaccharide derived from the exoskeleton of crustaceans and it is non-toxic, stable, biodegradable, and sterilizable [21,22]. Chitosan can be chemically modified with different stimulus-response moieties because of its molecular structure of D-glucosamine and N-acetylglucosamine (e.g., N-carboxyethyl chitosan (CEC) [23], glycol chitosan [24]). Moreover, chitosan demonstrates film-forming properties and is primarily utilized in food packing and biomedical applications [5]. Chitosan films exhibit good biocompatibility and antimicrobial property [25,26]. Upon adding conductive elements, chitosan films can be designed to possess an electrical conductivity near native tissues and regulate the differentiation of stem cells [2,8,27]. Therefore, chitosan along with conductive elements are anticipated to open a new frontier in wound healing and tissue engineering.

Recently, a hydrated conductive hydrogel/scaffold with three-dimensional porous structure and needle injectability was developed [28]. Conductive nanoparticles (i.e., double-bonded chitosan grafted polypyrrole, DCP) used in the earlier work showed good biocompatibility and considerable conductivity. However, such a hydrogel/scaffold can only be used under a hydrated state to present various properties. Most chitosan-based hydrogels, including the one published earlier [28], are brittle after drying, while conductive films need to keep favorable physical properties and biocompatibility in the dried state. Therefore, this is a new attempt to develop a biodegradable conductive film with low brittleness and coating possibility. In the present study, we aim to develop a multifunctional two-dimensional thin film combining DAPU nanoparticles, CEC, and DCP, which can be used under dry conditions and maintain adequate conductivity. Such thin films, crosslinking the two chitosan derivatives with DAPU, are obtained under room temperature and are expected to possess various functions such as strain sensing and thermal responsiveness. The potential functionality and application of the conductive thin film as a biocompatible polymer coating strategy are evaluated.

## 2. Materials and Methods

### 2.1. Materials

Poly(ε-caprolactone) diol (PCL, Mn 2000 Da, Sigma-Aldrich, St. Louis, MO, USA), isophorone diisocyanate (IPDI, Acros, Geel, Belgium), Sn(Oct)_2_ (Alfa Aesar, Haverhill, MA, USA), 2,2-bis(hydroxymethyl) propionic acid (DMPA, Sigma-Aldrich, St. Louis, MO, USA), triethylamine (TEA, J.T. Baker, Phillipsburg, NJ, USA), ethylenediamine (EDA, Tedia, Fairfield, OH, USA), glyoxal (Alfa Aesar, Haverhill, MA, USA), chitosan (170 kDa, 97% deacetylation, Kiotec, Hsinchu, Taiwan), acrylic acid (Showa, Gyoda, Japan), NaOH (Sigma-Aldrich, St. Louis, MO, USA), methacrylic anhydride (MAA, Sigma-Aldrich, St. Louis, MO, USA), acetic acid (Fluka, Buchs, Switzerland), pyrrole (Alfa Aesar, Haverhill, MA, USA), oxidant ammonium persulfate (APS, Sigma-Aldrich, St. Louis, MO, USA), HCl (Showa, Gyoda, Japan), high glucose Dulbecco’s modified Eagle’s medium (HG-DMEM, Gibco, Waltham, MA, USA), fetal bovine serum (FBS, Gibco, Waltham, MA, USA), Ham’s F-12 (Gibco, Waltham, MA, USA), G418 (Invitrogen, Waltham, MA, USA), penicillin-streptomycin-amphotericin (PSA, Caisson Laboratories, Inc., Smithfield, UT, USA), cell counting Kit-8 (CCK-8, Sigma-Aldrich, St. Louis, MO, USA), and KAPA SYBR Green qPCR kit (Kapa Biosystems, Inc., Wilmington, MA, USA) were applied in commercial form.

### 2.2. Waterborne Synthesis of Dialdehyde Polyurethane Crosslinker (DAPU)

DAPU was synthesized by the waterborne process through glyoxal termination of polyurethane in the final step of the polymerization, as detailed in our previous study [20]. For chemical synthesis, 10 g of PCL (soft segment) and 3 g of IPDI (hard segment) in a glass flask were reacted for 3 h with 0.05% catalyst Sn(Oct)_2_ at a nitrogen atmosphere of 75 °C. Mass of 0.6699 g of DMPA (hard segment) and appropriate methyl ethyl ketone solvent were put into the flask under reflux and reacted for another hour. Afterwards, 0.505 g of TEA was added at 50 °C and reacted for 30 min. Deionized distilled (DD) water was used to disperse the product followed by the reaction for 1 h with 0.21 g EDA (hard segment) as the chain extension agent. Finally, 0.5075 g of glyoxal was added and reacted for 30 min to form the dialdehyde polyurethane crosslinker dispersion. The stoichiometric ratio of oligodiol/IPDI/DMPA/EDA/glyoxal was 1:2.7:1:0.7:0.7.

### 2.3. Synthesis of N-Carboxyethyl Chitosan (CEC)

CEC was synthesized by functionalization of chitosan, as described in the previous literature [29]. To achieve the proper extent of functionalization, 4.0 g chitosan, 100 mL DD water, and 2.88 g acrylic acid were put into a flask and reacted at 50 °C for 48 h. The pH value of the CEC solution was adjusted to 7 with 1 M NaOH and dialyzed (12,000–14,000 MWCO) to remove unreacted substances or small molecules. Lastly, the CEC powder with a controlled degree of carboxyethyl functionalization was obtained by freeze-drying to remove water and stored at −20 °C. 

### 2.4. Synthesis of Polypyrrole Modified with Double-Bonded Chitosan (DCP)

The synthesis of nanoparticle DCP was based on the previous literature [28,30]. Chitosan was modified with MAA to produce double bond chitosan (DCS) as an intermediate. First of all, 2.14 g chitosan was slowly added to 50 mL of 2 wt% acetic acid. The chitosan solution was stirred for 2 h at 80 °C and then cooled down to 25 °C. A total of 185 μL aqueous MAA was added drop by drop to the chitosan solution and continuously stirred at a constant speed for 12 h, dialyzed with DD water, and then freeze-dried to get pure DCS solid. DCS and pyrrole were polymerized by APS to prepare DCP dispersion. DCS (100 mg) was dissolved in 25 mL 0.1 M HCl. Also, 578 μL pyrrole was added to the DCS solution and stirred for 2 h at room temperature. APS (0.19 g) was added into the system slowly for 24 h and was neutralized to pH = 7.4 by 1 M NaOH solution to obtain the final DCP dispersion. 

### 2.5. Preparation of DAPU/CEC/DCP Thin Films (DCDFs)

Various DCDFs were prepared by mixing CEC, DCP, and DAPU in the proper order to form a precursor. CEC aqueous solution should be mixed first with DCP, and then added with various concentrations of DAPU before vortex to obtain the uniform precursor. The precursor was poured into a Teflon dish (or other moulds) and removed of the remaining solvent at 25 °C to obtain the final DCDF.

### 2.6. Physico-Chemical Characterization of DCDFs

Surface contact angles were measured with an optical contact angle meter at ambient temperature. A sample (80 mm × 80 mm) was put on the movable sample stage and levelled horizontally. DD water droplets were dropped carefully onto the surfaces, the average value of five measurements at different positions of the sample was adopted as the contact angle, and the angle changes were recorded versus time. The pyrolytic temperatures of all thin films were obtained using thermogravimetric analysis (TGA, Q50, TA Instruments Inc., New Castle, DE, USA) at a heating rate of 10 °C/min under a nitrogen atmosphere. Each sample was 5 mg in weight. The melting temperature of various thin films was measured by differential scanning calorimetry (DSC, Q20, TA Instruments Inc., New Castle, DE, USA). Each sample was 5 mg in weight and was placed in an aluminum mold. The operating temperature was elevated from −80 °C to 160 °C at a rate of 5 °C/min under a nitrogen atmosphere. Melting temperatures (*T*_m_) were obtained at the peak of the melting endotherms, and the enthalpies of fusion (Δ*H*_m_) were obtained from the areas under the peaks. The crystallinity degree (*X*c) was determined by the equation Δ*H*_m_/ω·Δ*H*_m_^0^ × 100%, where Δ*H*_m_ is the experimental melting enthalpy and ω is the weight fraction of material [31]. Additionally, it was assumed that the enthalpy of melting (Δ*H*_m_^0^) of 100% crystalline PCL and chitosan was 139 J/g and 189 J/g, respectively [32]. The modulus of elasticity (Young’s modulus) of each group was calculated by the equation σ/ε, where σ was the tensile stress and ε was the axial strain. The surface profiler (Surfcorder ET3000) was used to measure the two-dimensional surface roughness of thin films. The images of the cross-section and surface from the thin films were acquired using a scanning electron microscope (SEM, Hitachi TM 3000, Hitachi, Ltd., Tokyo, Japan) operated at a voltage of 5 kV. The mechanical properties of the thin film were tested by a stretching machine and a dynamic mechanical analyzer (DMA, Q800, TA Instruments Inc., New Castle, DE, USA). Dogbone-shaped tensile specimens were cut from the internationally regulated dumbbell cutter. Tensile tests were performed on a Tensilon (World Meteorology CO., Ltd., Taichung City, Taiwan) tensile tester, and the samples were pulled at a rate of 1 mm/min under 25 °C and 50 °C. The DMA was used to analyze the modulus of thin films at a rate of 1 mm/min under 25 °C and 50 °C.

### 2.7. Strain Sensing Function of DCDFs

The sensitivity of DCDF to multiple strains, including compression, bending, and twisting, was studied using a single-channel system source meter (2601B, Keithley Instruments, Inc., Cleveland, OH, USA) to receive the conductivity through the four-point probe under a voltage of 0.5 V at 25 °C dried environment. All the samples were cut to a single size 25 mm × 10 mm × 0.5 mm (length × width × thickness). The compressive stress was applied at a weight of 50 g on DCDF. The bending strain was given to the DCDF upon repeated bending from 90° to 0°. With one end fixed, another end of the DCDF was twisted to the designed torsion angle 180° to record the twisting strain. The conductivity changes were calculated by the formula Δ*R*/*R*_0_ = (*R*_i_ − *R*_0_)/*R*_0_, where *R*_i_ represents the real-time conductivity and *R*_0_ represents the conductivity in the absence of strain [33].

### 2.8. Cell Attachment and Proliferation Analysis

Neural stem cells (NSCs) derived from the brain of adult mice were used for the cell culture test. The medium of NSCs was the HG-DMEM, which contained 10% FBS, Ham’s F-12, 400 mg/mL G418, and 1% PSA. NSCs were cultured in a humid incubator containing 5% CO_2_ at 37 °C, and the culture medium was refreshed every two days. DCDFs were irradiated with UV light for 24 h before inoculation of NSCs (cell density 4 × 10^4^ cells/film). The CCK-8 assay was used to evaluate the ability of cell proliferation on the thin film every day.

Time-lapse images were recorded by an ASTEC^®^ CCM-1.4XZY/CO_2_ system (ASTEC CO., Ltd., Fukuoka, Japan) with a CCD camera mounted on a time-lapse microscope with a magnification ratio of 100:1. The temperature for all screens was maintained at 37 °C. The image collection time took place from 0 h to 24 h after a pre-incubation period of 3 h.

### 2.9. Gene Expression of Neural-Related Marker for NSCs on DCDFs

The gene expression of NSCs was performed on DCDFs and control groups to observe the differentiation behavior of cells. The gene expression of neurological markers was analyzed by the KAPA SYBR Green qPCR kit through a quantitative reverse transcription-polymerase chain reaction (RT-PCR), and the data were collected by the StepOnePlus Real-Time PCR instrument (Applied Biosystems, Foster City, CA, USA). The neurological marker genes evaluated in this study were nestin, glial fibrillary acidic protein (GFAP), β-tubulin, and microtubule-associated protein 2 (MAP2). Glyceraldehyde 3-phosphate dehydrogenase (GAPDH) was used to standardize the gene expression levels in relative proportions.

### 2.10. Evaluation of DAPU/CEC/DCP (DCD) Materials as a Conductive Coating

Two methods were used to confirm that DCD materials may be employed as a polymer coating, including atmospheric plasma coating and overlay coating. The overlay coating was to directly coat the DCD precursor uniformly on the glass substrate. In the atmospheric plasma coating method, the surface of the substrate was first modified by scanning with air plasma, and then the DCD precursor was uniformly coated on the plasma-modified substrate. The plasma equipment was an open-air plasma system (Openair^®^) developed by Plasmatreat (Steinhagen, Germany). The plasma temperature at the nozzle exit was 25 °C and the air pressure was 2.5 kg/cm^2^. The frequency of plasma ejection was set to 21,000 Hz. The substrate was placed at a distance of 150 mm from the nozzle and the scanning speed of the nozzle was 30 cm/min. All the samples were dried at room temperature.

### 2.11. Statistical Analysis

All experimental results in this study were independently performed with at least three replicates to rule out contingency. The statistical differences between the experimental groups were performed by applying the commercially available statistical software package GraphPad Prism 4 and performed by two-way ANOVA with Tukey’s posthoc statistical testing. The quantitative data of this experiment are presented in the form of “mean ± standard deviation”. Data with *p* values smaller than 0.05 represented those with statistical significance.

## 3. Results and Discussion

### 3.1. Preparation and Optimization of DCDFs

**Figure 1:**
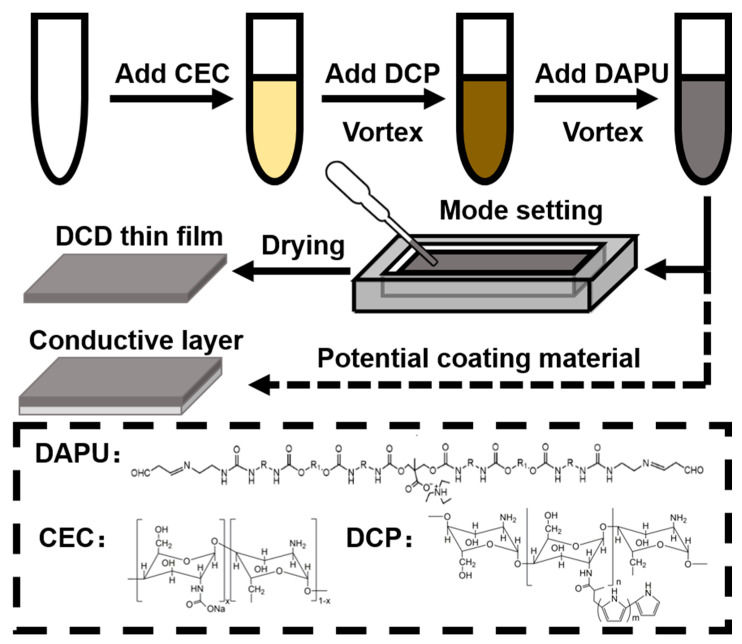
A schematic representation of the preparation process of the DAPU/CEC/DCP (DCD) thin films and their potential as biocompatible conductive polymer coating. DAPU: dialdehyde polyurethane; CEC: N-carboxyethyl chitosan; DCP: double-bonded chitosan modified polypyrrole.

Conductive DCDFs were prepared by adding the precursors (CEC, DCP, and DAPU) in proper order into a mould and dried at 25 °C, as demonstrated in Figure 1. The conductive DCDFs were expected as potential biocompatible conductive polymer coating materials (described later in Section 3.5). Various DCDFs were generated using different component ratios. The conductivity of DCDFs with different formulae was tested by the four-point probe. The roughness was evaluated using the profilometer. Results are summarized in Table 1. The conductivity of DCP-containing DCDFs, measured in the dried state, was significantly higher than the non-DCP-containing control film. Among the three DCDFs with different contents, DCDF2 showed the maximum value of conductivity (2.83 ± 0.17 mS/cm). In terms of roughness, all groups demonstrated rough surfaces, especially the control group without DCP. After optimization, the conductive thin films selected for further studies were those containing 49.75 wt% DAPU, 49.75 wt% CEC, and 0.50 wt% DCP (i.e., DCDF2 in Table 1). The notable differences of properties before the current materials and the materials from drying the earlier hydrogel may be associated with the low glass transition temperature (*T*_g_) of the soft segment of the materials. We hypothesize that when the components in DCDFs are crosslinked with the gradual removal of the moisture, the low *T*_g_ soft segment (PCL) of DAPU would migrate, resulting in chain entanglement and self-assembly among the nanoparticles. This entanglement and self-assembly of DAPU may give rise to lower steric hindrance and a higher crosslinking degree for the resulting films. In contrast, if the crosslinking occurs first as in the hydrogel, the steric barrier of the crosslinking network would interfere with the entanglement and self-assembly of PCL segments during the drying process. The less favorable microphase separation may also contribute to the brittleness of the dried hydrogel. Meanwhile, the adequate electrical conductivity remained under a dry state for regulating biological activities [34,35]. The different roughness between the control group and DCDFs could be attributed to the presence of DCP. The addition of the positively charged DCP may produce an ionic reaction with negatively charged CEC and negatively charged DAPU and facilitate the more homogeneous crosslinking for networks of DCDFs [36]. The control groups used in all the following experiments were the non-DCP-containing films.

**Figure 2:**
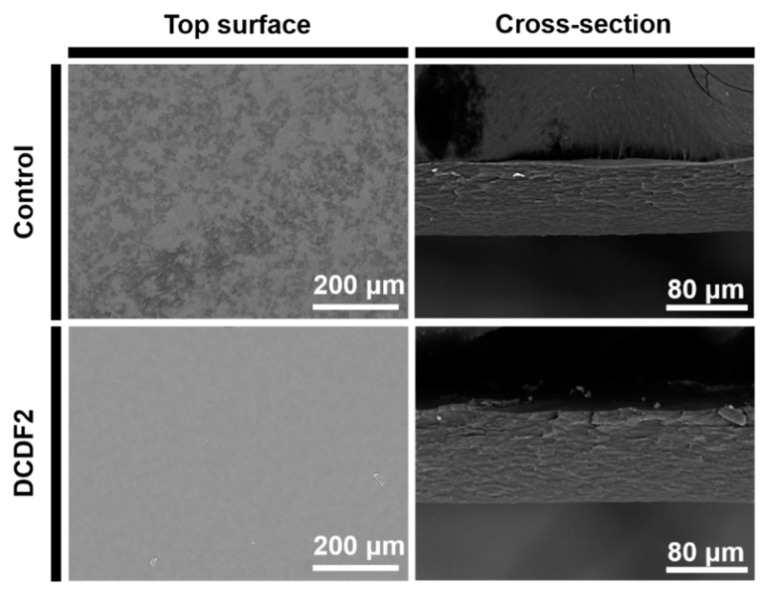
Scanning electron microscope (SEM) images of the control film and the DCDF film (DCDF2) in top-surface and cross-sectional views.

The surface topography and hydrophilicity, related to the roughness of DCDFs [37], were further evaluated. SEM images of the control group confirmed its high surface roughness, consistent with the results obtained by the profilometer. SEM images of DCDFs and the control group showed in Figure 2 and Figure S1 (Supplementary Materials) revealed that all films were dense films (i.e., the surface and cross-section of films were non-porous). The DCDFs were two-dimensional substrate and did not possess a three-dimensional porous structure such as that of the cryogel or scaffold. Meanwhile, the DCDF1 showed a rougher surface than the other two DCDF groups due to the abundance of CEC in the system, which was consistent with the previous literature [38]. 

**Figure 3:**
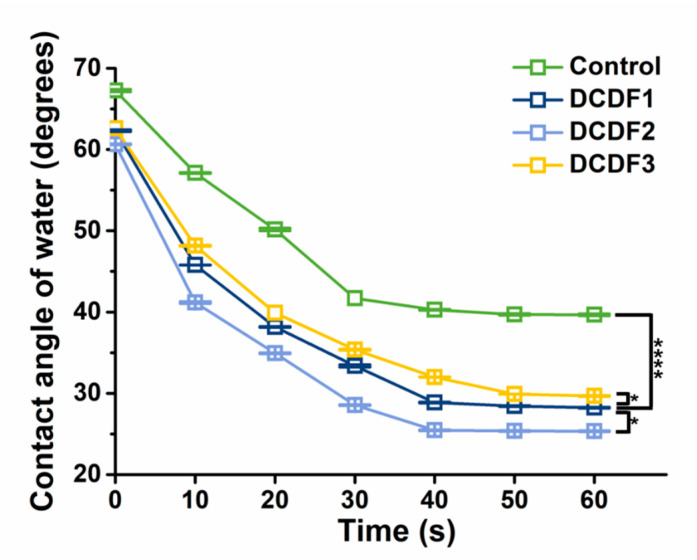
Water contact angles for the surface of the control group and different DCDFs as a function of time. The data points were an average of five test results. Control group: thin films without DCP. * *p* < 0.05 and **** *p* < 0.0001 between the indicated groups.

Analysis of the contact angle is one of the most established methods for investigating the surface properties of thin films. The contact angle takes into account the geometric measurement or the angle formed at the intersection of the liquid phase, gas phase, and solid phase, thus providing a direct evaluation of surface wettability [39]. The contact angle of water on the surface of DCDFs and the control group within the initial 60 s are shown in Figure 3. The contact angle of water on the surface of DCDFs dropped quickly from ~63° to around 27°, and the control group remained ~40° after 60 s, which means there was about a 25% decrease in the water contact angle because of the DCP addition. Significant differences existed between the contact angle of water on the surface of DCDF2 and the other groups. The pure chitosan film had significantly greater contact angle values (~90°) [40]. DCDF2 showed good hydrophilicity and had a low water contact angle (25.4°), close to that of the collagen surface [41]. These results showed that the conductive DCD materials may be a potential candidate for making hydrophilic conductive surface coatings.

### 3.2. Physico-Chemical Properties of Conductive DCDFs

**Figure 4:**
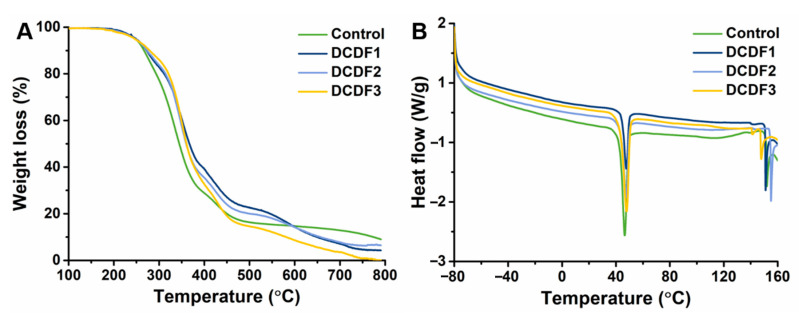
Thermal properties of the control group and DCDFs by (**A**) thermogravimetric analysis and (**B**) differential scanning calorimetry. Control group: thin films without DCP.

The thermal properties of DCDFs and the control group were evaluated by TGA and DSC. Results are shown in Figure 4, with the melting temperatures, enthalpies, and crystallinity of DCDFs summarized in Table 2. The TGA curves in Figure 4A indicated that all the thin films were stable at high temperatures up to about 170 °C. All the curves for DCDFs showed a slower decay than that of the control group in the temperature range from ~250 °C to ~320 °C, indicating that the slower decay was contributed to by the presence of DCP (Figure S2, Supplementary Materials). The DSC results demonstrated that all the films had a prominent endothermic peak at ~45 °C, probably associated with the crystallinity of PCL in the soft segment of the DAPU crosslinker [42]. There were two endothermic peaks (a small peak and a big sharp peak) between 140–160 °C in all films, probably associated with the crystallization of CEC [43]. The enthalpies of the control group, DCDF1, DCDF2, and DCDF3 from PCL were 11.03, 3.26, 8.86, and 8.03 J/g, respectively. In addition, the enthalpies of the control group, DCDF1, DCDF2, and DCDF3 from chitosan were 10.13, 8.32, 8.02, and 7.44 J/g, respectively. Furthermore, the crystallinity of PCL and chitosan in the DCDFs was estimated based on the results of DSC curves. The crystallinity of PCL in each group (i.e., control group, DCDF1, DCDF2, and DCDF3) was 22.67%, 6.70%, 18.21%, and 16.51%, respectively. The crystallinity of CEC in each group was 10.71%, 8.80%, 8.48%, and 7.87%, respectively. The control group showed the highest enthalpy and crystallinity for either the PCL soft segments of DAPU or CEC in the absence of DCP that may interfere with crystallization. The crystallinity of CEC was reduced with the decrease of CEC content in the DCDF system. Among three DCDFs, DCDF2 displayed the highest enthalpy and crystallinity for PCL soft segments of DAPU and CEC, probably due to the appropriate degree of crosslinking in the whole system. PCL, as the only soft segment in the system, accounts for about 35 wt% of the whole system. The hard domains in this system include CEC, DCP, and a part of DAPU, which can lead to microphase separation in the dry state. The obvious crystallization behavior of PCL in DAPU may play an important role in affecting the mechanical properties of DCDFs. Therefore, the properties of the films below (25 °C) and above (50 °C) the PCL melting temperature (at ~47 °C) were further evaluated by DMA to verify the temperature-dependent modulus changes of DCDFs.

**Figure 5:**
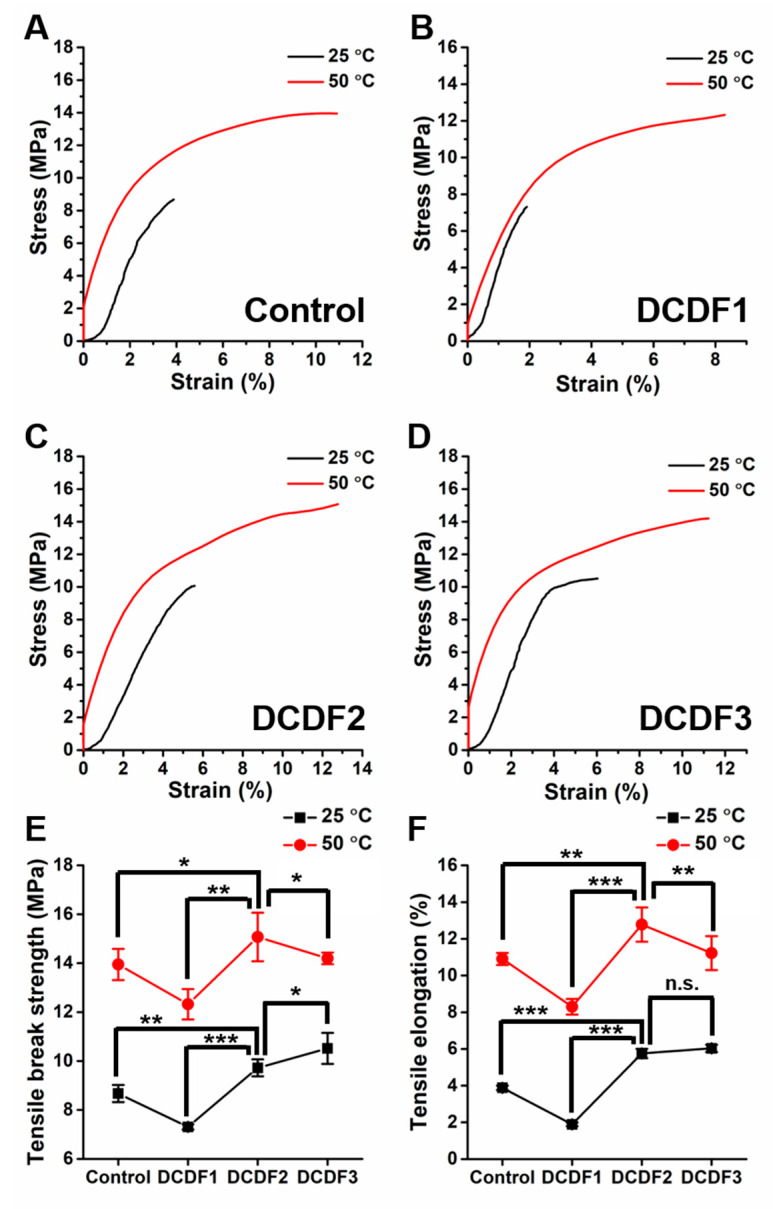
Tensile properties of DCDFs and the control group evaluated by DMA and a tensile tester under 25 °C or 50 °C. (**A**–**D**) Tensile stress-strain curves of (**A**) the control group, (**B**) DCDF1, (**C**) DCDF2, and (**D**) DCDF3. Changes of (**E**) tensile break strength and (**F**) tensile elongation of all films. * *p* < 0.05, ** *p* < 0.01, and *** *p* < 0.001 between the indicated groups.

The elastic tensile modulus of DCDFs versus strain was evaluated by DMA under 25 °C and 50 °C, respectively, as shown in Figure 5A–D. All thin films were also subjected to static tensile tests under the dry condition to obtain the values of tensile strength and elongation at break under 25 °C and 50 °C, respectively, as shown in Figure 5E,F. The modulus of elasticity of the control, DCDF1, DCDF2, and DCDF3 at 25 °C were 2.18 MPa, 3.17 MPa, 1.73 MPa, and 2.26 MPa, respectively. After heating to 50 °C, the modulus of elasticity in each group was decreased to a certain extent with the values of 1.83 MPa, 2.78 MPa, 1.52 MPa, and 1.79 MPa, possibly due to the melting of the soft segment (PCL). The data showed that addition of DCP improved the tensile break strength (from 8.67 MPa to 9.72 MPa) and elongation (from 3.88% to 5.76%) under 25 °C in the two groups with the same proportion of DAPU and CEC (the control group and DCDF2, m _CEC_: m _DAPU_ = 1:1). At the same time, increasing the amount of DAPU in the case of DCDF2 and DCDF3 slightly increased the tensile properties of DCDFs. There were significant differences between each experimental result of DCDF2 and those of the control and the DCDF1 group. After heating, there also existed significant differences in the tensile break strength and elongation between DCDF2 and DCDF3. Since chitosan and its derivatives are brittle in the dry state, abundant CEC may cause phase separation in the dry DCDF. The tensile properties of such thin films may be lower due to stress concentration after stretching (as compared to DCDF1). Accordingly, unreacted DAPU, as an elastomer, may serve as a reinforcing agent to enhance the tensile properties of DCDFs below the PCL crystallization temperature. When the temperature was heated above the crystallization temperature of PCL (~50 °C), the tensile break strength (9.72 MPa to 15.07 MPa) and the tensile elongation (from 5.76% to 12.77%) of DCDF2 were obviously increased. Although the tensile properties of each group were improved at the higher temperature, DCDF2 remained to possess the best tensile properties at both 25 °C and 50 °C. PCL, as the soft segment composition of DAPU, would soften when the temperature reached above 50 °C, which enhanced the stretchability of DCDFs. The hard and soft segments of DAPU may undergo structure and phase rearrangement when the film was stretched, leading to the increase of tensile strength. The tensile properties of DCDF1 and DCDF3 were not as good as that of DCDF2, probably because the phase separation between the components was less favorable for rearrangement during the stretching process. Meanwhile, DCDF2 had the higher crystallinity of PCL segments than DCDF1 and DCDF3, which may also be the reason for better tensile properties after heating above 50 °C.

### 3.3. Strain Sensing Functions of DCDFs

**Figure 6:**
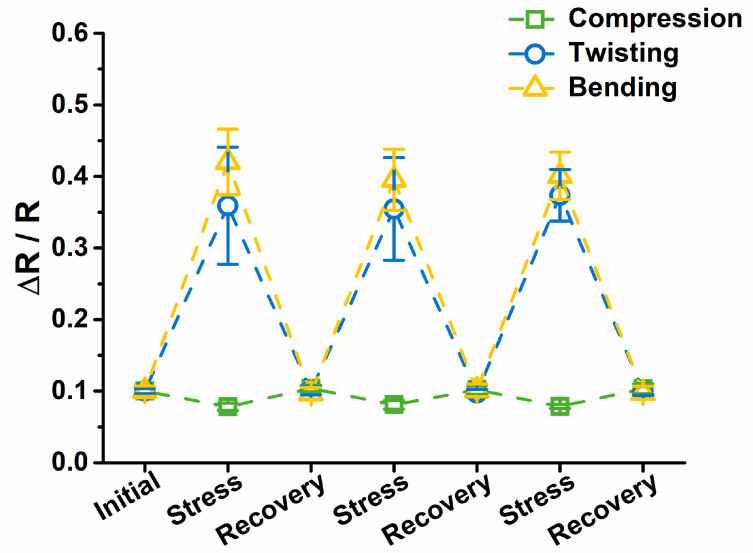
Sensing functions of DCDFs under 0.5 V of applied voltage. Changes of the thin film conductivity versus repeated loading weight of 50 g, repeated twisting of 180°, and repeated bending of 90°. Data were obtained from DCDF2.

The conductive DCDFs showed sensitivity to strains of various modes, including compression, twisting, and bending, as shown in Figure 6. The conductivity variation of the dried DCDFs was collected by a single-channel system source meter. The compressive strain was given by repeated loading of the weight (50 g). The conductivity variation of the film only slightly decreased after loading the weight (~0.1 to ~0.08). The response of DCDFs to compressive strain was not obvious, which may be limited by the thickness of the film. The conductivity variation was more obvious for the twisting mode (from ~0.1 to ~0.36) or the bending mode (from ~0.1 to ~0.40). The conductivity changes during the repeated deformation were all reproducible and reversible (i.e., returning to the initial value after strain removal) [44]. These data, together with the biocompatibility that will be discussed later, suggest the potential of DCDFs as wearable or implantable flexible human motion-detective sensors.

### 3.4. Cell Morphology, Proliferation, and Differentiation of NSCs on DCDFs

**Figure 7:**
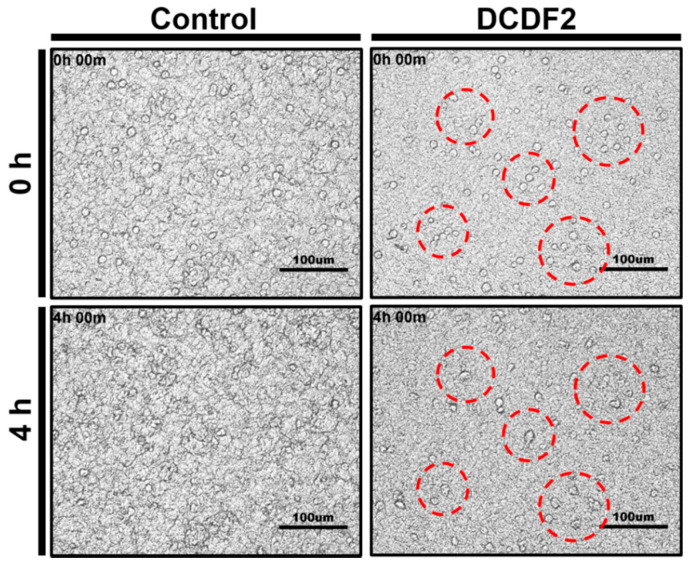
The morphology of neural stem cells (NSCs) seeded on DCDFs examined using time-lapse recording after pre-incubation for 3 h. NSCs were seeded in 12-well plates (4 × 10^5^ NSCs per well) coated with DCDFs or the control group. The NSCs on DCDFs had obvious aggregation behavior after 4 h, indicated by red circles. Data were obtained from DCDF2. Control group: thin films without DCP.

To confirm NSCs were attached on DCDFs, the cell morphology of NSCs after pre-incubation for 3 h was constantly monitored using time-lapse recording. During the 24-h recording, NSCs were found to attach on both DCDFs and the control group (Figure 7) with continuous changes of cell morphology (Movie S1, Supplementary Materials). Meanwhile, the recording showed that NSCs on DCDFs had obvious clustering behavior after 4 h and tended to develop into multicellular spheroids, but this clustering phenomenon was not seen in the control group. The multicellular spheroids of NSCs have been reported to mimic the neurospheres and may favor neural differentiation [24,45].

**Figure 8:**
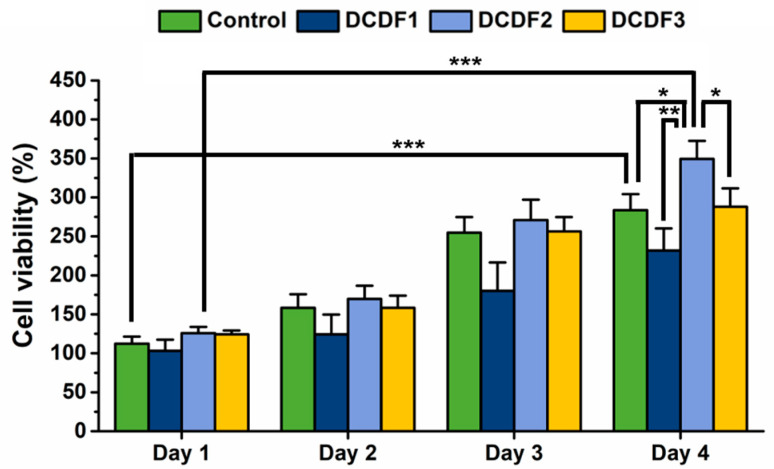
The proliferation of neural stem cells (NSCs) on DCDFs in a period of 4 days compared with the control group (cells on the thin film without DCP). The data were obtained using the CCK-8 assay. The viability of NSCs was deducted from that of the blank group (the same thin films without cells), normalized to the value at day 0, and expressed as the percentage of cell viability (%). * *p* < 0.05, ** *p* < 0.01, and *** *p* < 0.001 between the indicated groups.

Proliferation of NSCs on DCDFs in a period of 4 days was compared with the control group. The data were obtained using the CCK-8 assay, as shown in Figure 8. After 4 days, the cell viability was ~329.3% and ~283.7% for DCDF2 and the control group (without DCP), respectively. On the other hand, the cell viability on DCDF1 and DCDF3 was ~231.9% and ~288.0% at 4 days, lower than that on the DCDF2 (~329.3%). There were significantly differences in the viability of NSCs on DCDF2 from the other groups at 4 days.

**Figure 9:**
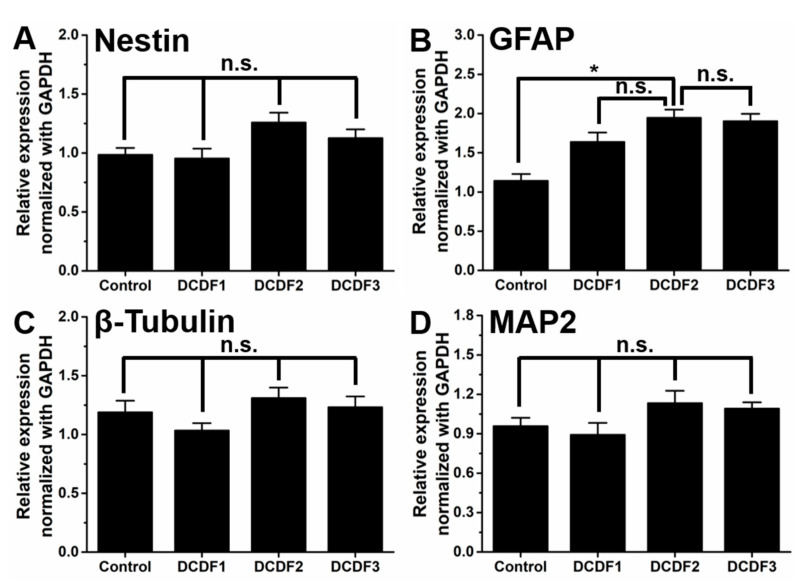
The differentiation of NSCs on DCDFs. The gene expressions of neural-related genes, including (**A**) Nestin, (**B**) GFAP, (**C**) β-tubulin, and (**D**) MAP2, were analyzed by RT-PCR at 4 days. The expression levels are represented by the relative ratios of gene expression normalized to that of GAPDH. * *p* < 0.05 between the indicated groups. Control group: thin films without DCP.

The gene expression of NSCs was performed on DCDFs and the non-DCP-containing control after 4 days to verify the cell differentiation behavior. The expression of neural-related genes, including nestin, GFAP, β-tubulin, and MAP2, is displayed in Figure 9, where the expression was normalized to GAPDH gene and then expressed in relative proportions. The expression levels of nestin (stemness marker), β-tubulin (early neuronal marker), and MAP2 (mature neuronal marker) genes after 4 days showed no significant differences between the DCDFs and the control group. In contrast, the expression level of the GFAP (glial marker) gene was significantly upregulated for NSCs on DCDF2 after 4 days as compared to the control group, but there was no significant difference among the control, DCDF1, and DCDF3. According to previous literature [46,47], chitosan-based materials promoted the survival of stem cells and neurite extension owing to the degradation products of chitosan. Compared to the conductive hydrogel [28], two-dimensional conductive DCDFs for 4 days seemed to promote the expression of the GFAP gene only, but not the other three related genes. The finding agreed with the literature reporting that two-dimensional polypyrrole-containing conductive films promoted the expression of glial-related genes (i.e., GFAP) more than the neuronal-related genes [48]. Taken together, these data suggested that DCDFs facilitated the aggregation, proliferation, and differentiation of NSCs, especially the DCDF2.

### 3.5. The Potential as Conductive Coating Materials

The potential of DCDFs employed as a polymer coating layer was demonstrated by two commonly used coating methods, including atmospheric plasma coating and overlay coating, as shown in Figure S3 (Supplementary Materials). As a coating material, the appropriate viscosity before coating and homogeneity after coating should be considered. Most published chitosan-based hydrogels cannot be dried and used as thin films due to their brittleness and excessive phase separation in the dry state. Because of the difficulty in coating chitosan-based hydrogels and casting them as dried films, not much research has been conducted on the related topic. DCD precursors, with adequate viscosity and homogeneity, are expected to have coating applications and produce multifunctional dried thin films. To start with, DCD materials were preliminarily coated on the 12-well plates to investigate the cell proliferation and differentiation (described in Section 3.4). In addition, some conventional coating methods (i.e., atmospheric plasma coating and overlay coating) were used to evaluate the potential as a conductive hydrophilic biocompatible coating material. The results showed that DCD materials can effectively and uniformly modify the surface of the substrate. Meanwhile, with reference to all above characteristics, the surface modification with DCD materials can endow both conductivity and hydrophilicity to the substrate, as well as enhance the cellular compatibility. This conductive DCD thin film/coating, to the best of our knowledge, is the first report on multifunctional film materials with hydrophilicity, thermal responsiveness, strain sensing, and biocompatibility, suggesting a promising surface coating candidate for biomedical applications.

## 4. Conclusions

Thermoresponsive and conductive thin films composed of DAPU, CEC, and DCP were successfully synthesized and prepared. The tri-component DCDFs showed hydrophilicity and densification. These thin films exhibited adequate conductivity (all above 2.50 mS/cm) in a dry state. Thermoresponsive tensile properties, i.e., tensile break strength (an increase from 9.72 MPa to 15.07 MPa) and tensile elongation (an increase from 5.76% to 12.77%), were verified by DMA under 25 °C or 50 °C. The potential for strain sensing was demonstrated by the relative changes of conductivity in response to the deformation of various modes. Besides, NSCs seeded on the surface of DCDFs showed greater cell proliferation and differentiation than those on non-conductive controls. Moreover, DCD materials may serve as a polymer coating. These findings suggested that thermoresponsive and conductive DCDFs developed in this study may be potential films and possible coating with hydrophilicity, thermal responsiveness, strain sensing, and biocompatibility.

## Figures and Tables

**Table 1:** The basic properties and abbreviated names for the conductive DAPU/CEC/DCP (DCD) thin films (DCDFs) prepared with different formulae. DAPU: dialdehyde polyurethane; CEC: N-carboxyethyl chitosan; DCP: double-bonded chitosan modified polypyrrole.

Abbreviation	DAPU/wt %	CEC/wt %	DCP/wt %	Conductivity/mS·cm^−1^	Roughness/μm
Control	50.00	50.00	0	0.11 ± 0.01	7.94 ± 0.54
DCDF1	45.21	54.25	0.54	2.64 ± 0.18	8.05 ± 0.36
DCDF2	49.75	49.75	0.50	2.83 ± 0.17	7.94 ± 0.23
DCDF3	56.90	42.67	0.43	2.54 ± 0.15	8.40 ± 0.28

**Table 2:** The detailed thermal properties of DCDFs summarized from differential scanning calorimetry (DSC) results.

Samples	Melting Temperature (*T*_m_)/°C			Enthalpy (Δ*H*_m_)/J·g^−1^		Crystallinity (*X*_c_)	
	PCL	CEC		PCL	CEC	PCL	CEC
Control	46.47	139.53	151.98	11.03	10.13	22.67%	10.71%
DCDF1	47.72	142.65	151.07	3.26	8.32	6.70%	8.80%
DCDF2	47.67	144.00	155.08	8.86	8.02	18.21%	8.48%
DCDF3	47.90	141.25	147.76	8.03	7.44	16.51%	7.87%

## Data Availability

Data sharing is not applicable to this article.

## Supplementary Materials

The following are available online at https://www.mdpi.com/2073-4360/13/3/326/s1. Figure S1: SEM images of DCDF1 and DCDF3 in top surface and cross-sectional view. Figure S2: The TGA curve of DCP. Figure S3: Testing of DCDFs as a potential coating. Movie S1: Cell morphology. Continuous changes of cell morphology and the movement of NSCs on the DCD thin film within 24 h.
