# Supplementary material for: Thermoresponsive and Conductive Chitosan-Polyurethane Biocompatible Thin Films with Potential Coating Application
Source: Polymers (Basel). 2021 Jan 20;13(3):326. doi: 10.3390/polym13030326 (PMC7864029; doi:10.3390/polym13030326)
Supplement: Supplementary file 1 [file polymers-13-00326-s001.zip › Supplementary Information-Polymers/Supplementary Information-Polymers.docx]

**Supplementary Information**

**Thermoresponsive and conductive chitosan-polyurethane biocompatible thin films with potential coating application**

Junpeng Xu ^1^, Chih-Yu Fu ^1^, Yu-Liang Tsai ^1^, Chui-Wei Wong ^1^, Shan-hui Hsu ^1,2,*^

^1^ Institute of Polymer Science and Engineering, National Taiwan University, No. 1, Sec. 4 Roosevelt Road, Taipei 10617, Taiwan, Republic of China

^2^ Institute of Cellular and System Medicine, National Health Research Institutes, No. 35 Keyan Road, Miaoli 35053, Taiwan, Republic of China

Pages: 3

Supplemental figures: 3 (Figure S1 – S3)

Supplemental videos: 1 (Movies S1)

* Corresponding author: Shan-hui Hsu

Institute of Polymer Science and Engineering, National Taiwan University, No. 1, Sec. 4 Roosevelt Road, Taipei 10617, Taiwan, R.O.C.;

Phone: +886-2-3366-5313;

Fax: +886-2-3366-5237;

E-mail: [shhsu@ntu.edu.tw](mailto:shhsu@ntu.edu.tw)

**
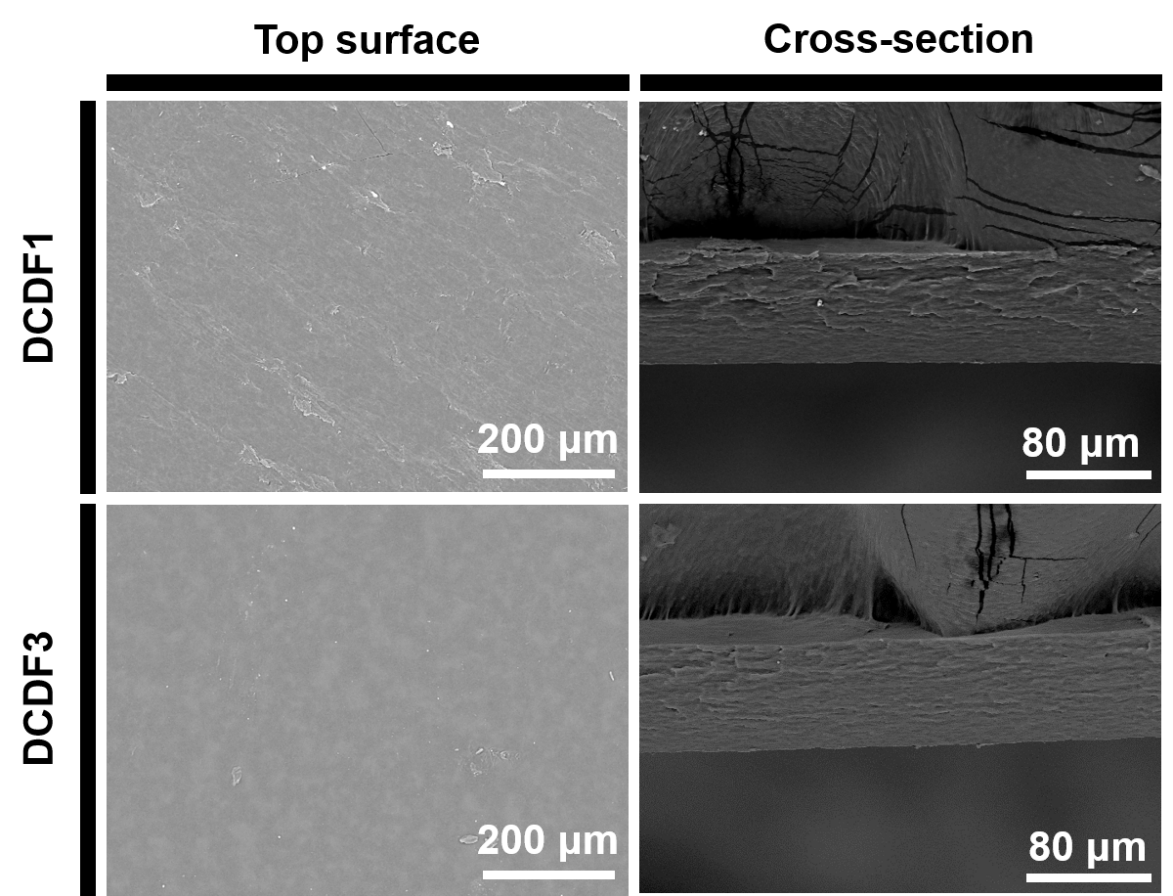
**

**Figure S1.** SEM images of DCDF1 and DCDF3 in top surface and cross-sectional view.





**Figure S2.** The TGA curve of DCP.

**
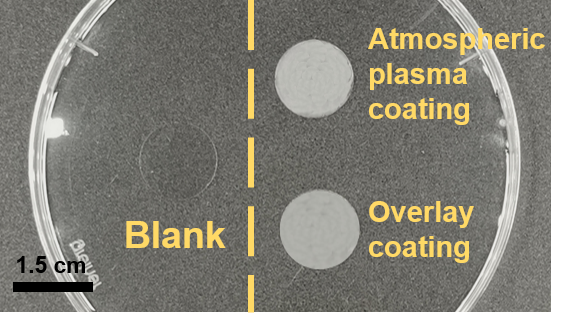
**

**Figure S3.** Testing of DCDFs as potential coating.

**Movie S1. Cell morphology.** Continuous changes of cell morphology and the movement of NSCs on the DCD thin film within 24 h.
